# Osteoarthritic and non-osteoarthritic patients show comparable coronal knee joint line orientations in a cross-sectional study based on 3D reconstructed CT images

**DOI:** 10.1007/s00167-021-06740-3

**Published:** 2021-09-26

**Authors:** Silvan Hess, Lukas B. Moser, Emma L. Robertson, Henrik Behrend, Felix Amsler, Edna Iordache, Vincent Leclercq, Michael T. Hirschmann

**Affiliations:** 1 Department of Orthopaedic Surgery and Traumatology, Kantonsspital Baselland (Bruderholz, Liestal, Laufen), 4101 Bruderholz, Switzerland; 2 Department of Orthopaedic Surgery and Traumatology, Kantonsspital St. Gallen, 9007 St. Gallen, Switzerland; 3 University of Basel, Basel, Switzerland; 4 Amsler Consulting, Basel, Switzerland; 5 Symbios, Yverdon les Bains, Switzerland

**Keywords:** Knee, Phenotypes, Distal femoral angle, Proximal tibial angle, Hip-knee-ankle angle, Osteoarthritis, Coronal alignment, Total knee arthroplasty

## Abstract

**Purpose:**

Recently introduced total knee arthroplasty (TKA) alignment strategies aim to restore the pre-arthritic alignment of an individual patient. The native alignment of a patient can only be restored with detailed knowledge about the native and osteoarthritic alignment as well as differences between them. The first aim of this study was to assess the alignment of a large series of osteoarthritic (OA) knees and investigate whether femoral and tibial joint lines vary within patients with the same overall lower limb alignment. The secondary aim was to compare the alignment of OA patients to the previously published data of non-OA patients. This information could be useful for surgeons considering implementing one of the new alignment concepts.

**Material:**

Coronal alignment parameters of 2692 knee OA patients were measured based on 3D reconstructed CT data using a validated planning software (Knee-PLAN^®^, Symbios, Yverdon les Bains, Switzerland). Based on these measurements, patients' coronal alignment was phenotyped according to the functional knee phenotype concept. These phenotypes represent an alignment variation of either the overall alignment, the femoral joint line orientation or the tibial joint line orientation. Each phenotype is defined by a specific mean and covers a range of ± 1.5° from this mean. Mean values and distribution among the phenotypes are presented and compared between two populations (OA patients of this study and non-OA patients of a previously published study) as well as between HKA subgroups (varus, valgus and neutral) using *t* tests and Chi-square tests (*p* < 0.05).

**Results:**

Femoral and tibial joint lines varied within patients with the same overall lower limb alignment. A total of 162 functional knee phenotypes were found (119 males, 136 females and 94 mutual phenotypes). Mean values differed between the OA and non-OA population, but differences were small (< 2°) except for the overall alignment (e.g. HKA). The distribution of OA and non-OA patients among the phenotypes differed significantly, especially among the limb phenotypes.

**Conclusion:**

Differences between OA and non-OA knees are small regarding coronal femoral and tibial joint line orientation. Femoral and tibial joint line orientation of osteoarthritic patients can, therefore, be used to estimate their native coronal alignment and plan an individualized knee alignment.

**Level of clinical evidence:**

III.

## Introduction

Correct orientation of the prosthetic components in total knee arthroplasty (TKA) is key to achieve satisfying functional outcomes. However, the dogma that a mechanically neutrally aligned prosthesis with a straight lower limb is optimal for all patients is increasingly questioned [1, 3, 11, 16, 20]. New individual TKA alignment strategies have been proposed, which aim to improve clinical outcomes by restoring the pre-arthritic alignment of the individual patient [1, 11, 12]. Clinical results of these concepts are promising, yet there are still unanswered questions [5, 11, 13]. The native alignment of a patient can only be restored with detailed knowledge about the native and osteoarthritic alignment as well as differences between them. Various studies highlighted the variability of the native alignment and contradicted the assumption that only a straight leg is physiological. Most recently, our understanding was further advanced by a study, which showed that femoral and tibial joint lines vary within young non-OA patients with the same overall lower limb alignment [8–10]. The current practice of categorizing patients by their overall alignment (i.e. “varus patients”) seems thus short-sighted, and the orientation of the joint lines should be included in the discussion. A new classification system based on phenotypes was proposed to facilitate a more detailed discussion. Until now, this classification system has not yet been applied to OA patients and the findings by Hirschmann et al. have not been confirmed in OA patients. Furthermore, our knowledge regarding differences between OA and non-OA patients is limited to conventional radiographs or/and studies with small sample sizes. The purpose of this investigation was thus the following: first, to apply the functional knee phenotype system to OA patients and investigate whether femoral and tibial joint lines vary within OA patients with the same overall lower limb alignment. Secondly, to assess differences between OA and non-OA patients with regard to overall lower limb alignment as well as tibial and femoral joint line orientation. It was hypothesized that in OA patients femoral and tibial joint line orientation vary within patients with the same overall alignment and that OA and non-OA knees differ with regard to mean values and distribution among the phenotypes. Based on the results of this analysis a hypothetical alignment concept will be applied to the OA population as example for the usage of the functional knee phenotype system. Understanding these differences and the impact of a system based on these differences could be useful for surgeons when assessing new alignment concepts. This will be the first study to implement the functional knee phenotype system in OA patients based on CT images and compare non-OA and OA patients based on this system.

## Material and methods

For this cross-sectional study we compared two patient populations. The OA population was based on the Knee-PLAN^®^ 3D database (Symbios Orthopédie S.A., Yverdon-Les-Bains, Switzerland), and the non-OA population was based on the hospital registry of the Kantonsspital Baselland (Kantonsspital Baselland, Bruderholz, Liestal, Laufen, Switzerland). Alignment parameters of the later have already been published [8–10].

The study was approved by the local ethical committee (Ethikkommission Nordwest- und Zentralschweiz (EKNZ), Nr. 2018-00223). All procedures performed were in accordance with the ethical standards of the institutional and/or national research committee and with the 1964 Declaration of Helsinki and its later amendments or comparable ethical standards.

### OA population

All CT scans collected between January 2017 and December 2019 in the Knee-PLAN® (Symbios Orthopédie S.A., Yverdon-Les-Bains, Switzerland) 3D database were retrospectively assessed and included if the following criteria were met: patients older than 50 and younger than 91 years at the time of imaging and no signs of previous fractures, osteotomies or rheumatoid arthritis. Patients were further excluded if a flexion deficit of more than 15° was present. A total of 2692 patients were finally included (1397 right and 1295 left lower limbs). The male to female ratio was 1075 (40%): 1617 (60%), and the overall mean age ± standard deviation (SD) was 71.1 ± 8.5 years (range 50–90 years).

### Non-OA population (previously published data)

The hospital registry (Kantonsspital Baselland, Bruderholz, Liestal, Laufen, Switzerland) was searched for patients aged between 45 and 16 years, who received a CT of the lower limb, according to the Imperial Knee Protocol [7]. Lower limbs were excluded for the following reasons: hip, knee or ankle prosthesis, osteotomy around the knee, any radiological signs of osteoarthritis or fractures and reported injury of the collateral ligaments. For reasons of consistency additional exclusion criteria were applied post hoc and 50 patients from the original cohort had to be excluded (all having more than 15° of knee flexion deficit during the CT scan). The resulting cohort consisted of 258 knees of 141 patients (90 males and 46 females, mean age ± SD 29.9 ± 6.4 years). Both legs from 117 patients and a single leg from 24 patients were included. SPECT/CT according to the imperial knee protocol was conducted for the following reasons: knee pain of unknown origin (e.g., anterior knee pain without trauma) (*n* = 22); osteochondritis dissecans (*n* = 13); persistent pain after treatment of sport injury (*n* = 87); and idiopathic patellar pathology (*n* = 0). A more detailed description of the population and its selection process was previously published [8].

### Alignment measurements and phenotyping

**Fig. 1:**
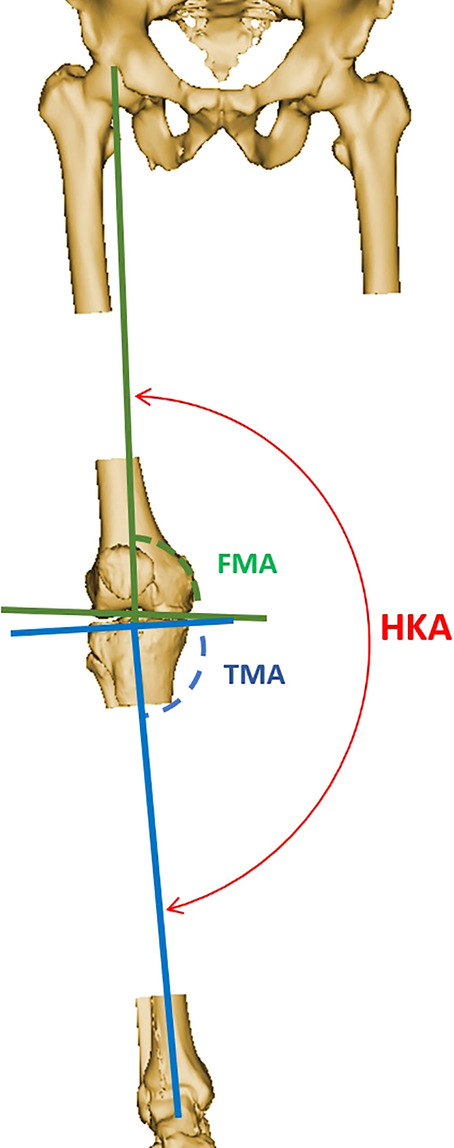
Lower limb alignment parameters: hip-knee-ankle angle (HKA), formed by the lines connecting the centres of the femoral head, the knee and the talus, representing the overall lower limb alignment. Femoral mechanical angle (FMA) between the femoral mechanical axis and a tangent to the distal femoral condyles, representing the orientation of the femoral joint line. Tibial mechanical angle (TMA) between the tibial mechanical axis and a tangent to the proximal tibia joint surface, representing the orientation of the tibial joint line

The following coronal alignment parameters were measured using a validated planning software (Knee-PLAN®, Symbios, Yverdon les Bains, Switzerland): hip-knee-ankle angle (HKA), femoral mechanical angle (FMA) and tibial mechanical angle (TMA). For reasons of consistency, the medial angle was always reported. Thus, an angle of less than 90° was considered as varus and an angle of more than 90° as valgus. Figure 1 shows a definition of all angles. All measurements were taken by trained engineers of the company with several years of experience in this field. For CE marking, the accuracy of measurements including inter- and intra-observer reliability has been reported as excellent, having measurement variability within 1° [6].

Based on these measurements, the alignment of all patients was phenotyped according to the previously introduced functional knee phenotype concept, which has been described elsewhere [8–10]. A phenotype represents a variation of either the overall lower limb (based on HKA) alignment, the femoral joint line orientation (based on FMA) or the tibial joint line orientation (based on TMA). An alignment variation (i.e. phenotype) is defined as a 3° range (1.5° + 1.5°) of one of these angles. The mean values of these phenotypes represent 3° increments of the angle starting from the overall mean value of the angle found in a young non-OA population (NEU_HKA_0° = 180°, NEU_FMA_0° = 93° and NEU_TMA_0° = 87°) [8, 9]. Accordingly, the neutral femoral phenotype ranges from 91.5° to 94.5° and the neutral tibial phenotype ranges from 85.5° to 88.5° and not from 88.5° to 91.5°. Knee phenotypes, which represent the joint line orientation of a patient, are formed by combining femoral and tibial phenotypes. Functional knee phenotypes are formed by combining all three phenotypes (limb, femoral and tibial), and they represent all aspects of the coronal alignment of a patient. The nomenclature of the phenotypes is organised as follows: the first part (NEU, VAR, VAL) defines the direction of alignment. The second subscripted part (HKA, FMA and TMA) states the measured angle. The last part (0°, 3°, 6°, etc.) shows the mean deviation of the phenotype from the mean value.

### Alignment concept

TKA alignment targets for every patient were defined according to the functional knee phenotype system and the following findings: (Nr. 1) patients with a preoperative varus overall alignment report better outcomes if their postoperative overall alignment is in varus as well [20], (Nr. 2) patients with a preoperative varus overall alignment report worse outcomes if their postoperative alignment is in valgus (vice versa for valgus patients), (Nr. 3) FMA and TMA do not change more than 2° during OA, (Nr. 4) angles should always be adjusted towards mechanically neutral and (Nr. 5) current TKA prosthesis should not be implanted outside certain safe zones (HKA limited to 175.5° to 184.5°, FMA limited to 88.5°–94.5°, TMA limited to 86.5°–91.5°). The resulting postoperative alignment for all OA patients was calculated based on the equation by Cook et al. [4]. Thereafter patients were excluded, if their alignment would change from varus to valgus or vice versa since these patients represent a special group.

The functional knee phenotype was used to define alignment targets because it incorporates safe zones and is thus applicable using conventional instrumentation. As an example, for patients with a preoperative FMA between 93.0 and 94.5° (i.e. measured FMA of 93.8°), the intraoperative target would be 93.0° (e.g. the mean of the femoral phenotype NEU_FMA_0°), resulting in a postoperative FMA of 93 ± 1.5° (based on a the assumption that a precision of about 1.5° is possible with conventional instrumentation). However, for some patients the lower board of the targeted phenotype was defined as intraoperative target to respect rule Nr 4 (“angles should always be adjusted towards mechanically neutral”). As an example, for patients with a preoperative FMA between 91.5° and 93° (i.e. 92.5), the intraoperative target would be 91.5° (e.g. the lower boarder of the femoral phenotype NEU_FMA_0°) resulting in a postoperative FMA of 91.5 ± 1.5° (based on the assumption that a precision of about 1.5° is possible with conventional instrumentation).

### Statistical analysis

A professional and experienced statistician performed all calculations. Descriptive statistics such as means, standard deviations, medians, ranges, and measures of variance are presented. *T* tests for independent samples were used to compare group differences, and coefficients of determination regarding the statistical strength of the relationships (*r*^2^) are shown. Mean values of all angles were compared between the two populations (OA patients of the present study and non-OA patients of a previously published study) as well as between subgroups (varus, valgus and neutral) of each population using *t* tests (*p* < 0.05). To test for the differences of deviations, Levene’s tests for homogeneity of variances were calculated. The different distributions of phenotypes were tested with Chi-square tests. The statistical level of significance was *p* < 0.05.

## Results

### Basic alignment parameters

Table 1 shows the mean values ± SD and the ranges found in the male and female OA population.

**Table 1:** Mean values ± SD and the ranges found in the male and female OA population

	Males (*n* = 1075)				Females (*n* = 1617)				Comparison		
Angle	Mean	SD	Min	Max	Mean	SD	Min	Max	Diff	*p*-value	*R*^2^
Hip-knee-ankle angle (HKA)	175.2	5	160	190	177.6	6	157	195	2.4	< 0.001	0
Femoral mechanical angle (FMA)	91.9	3	82.7	100	93.2	3	81	104	1.3	< 0.001	0.1
Tibial mechanical angle (TMA)	85.7	3	71	94	86.6	3	72	94.4	0.9	< 0.001	0

### Limb, tibial and femoral phenotypes

**Fig. 2:**
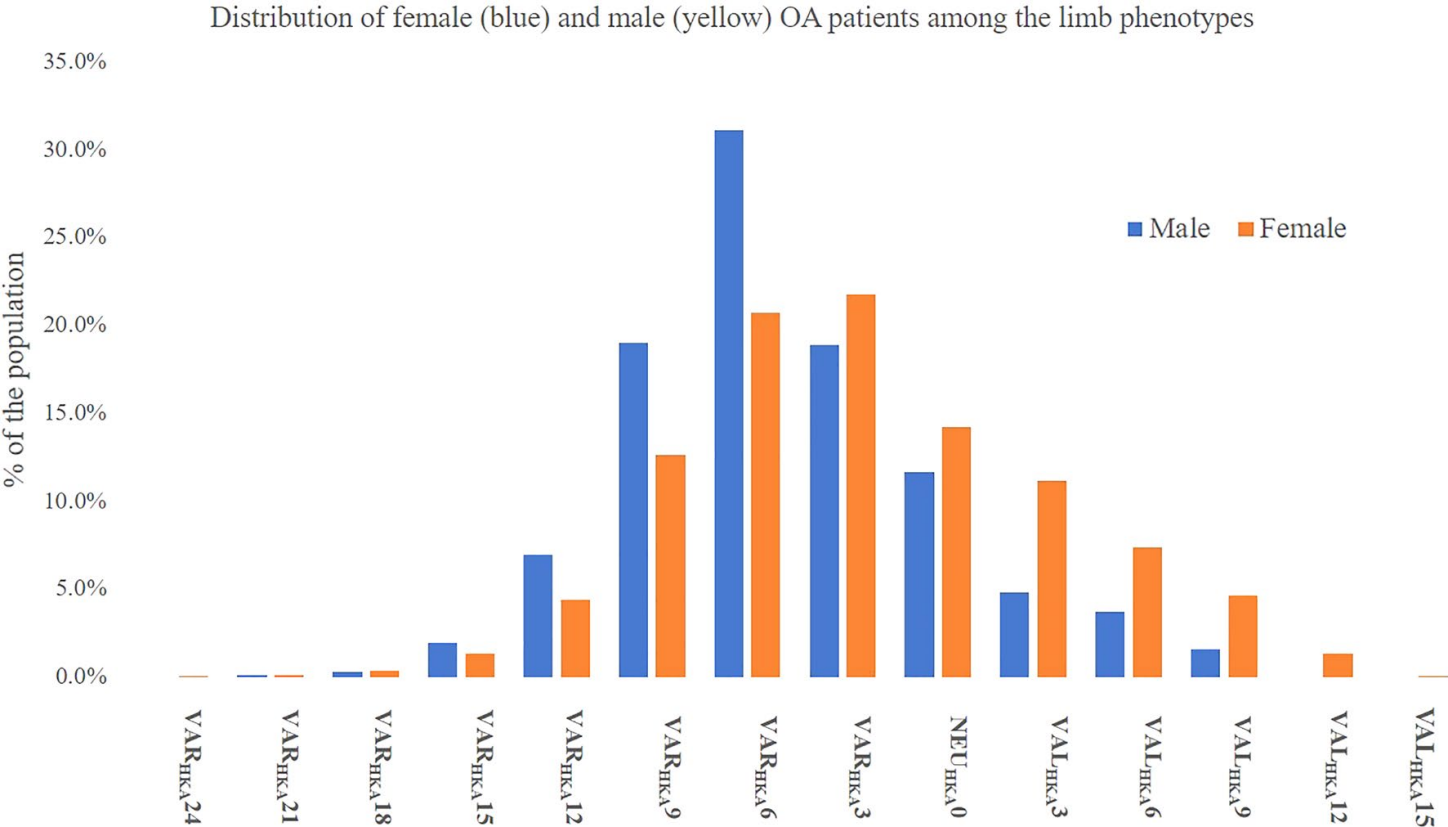
Distribution of the female (orange) and male (blue) population among the limb phenotypes in %

**Fig. 3:**
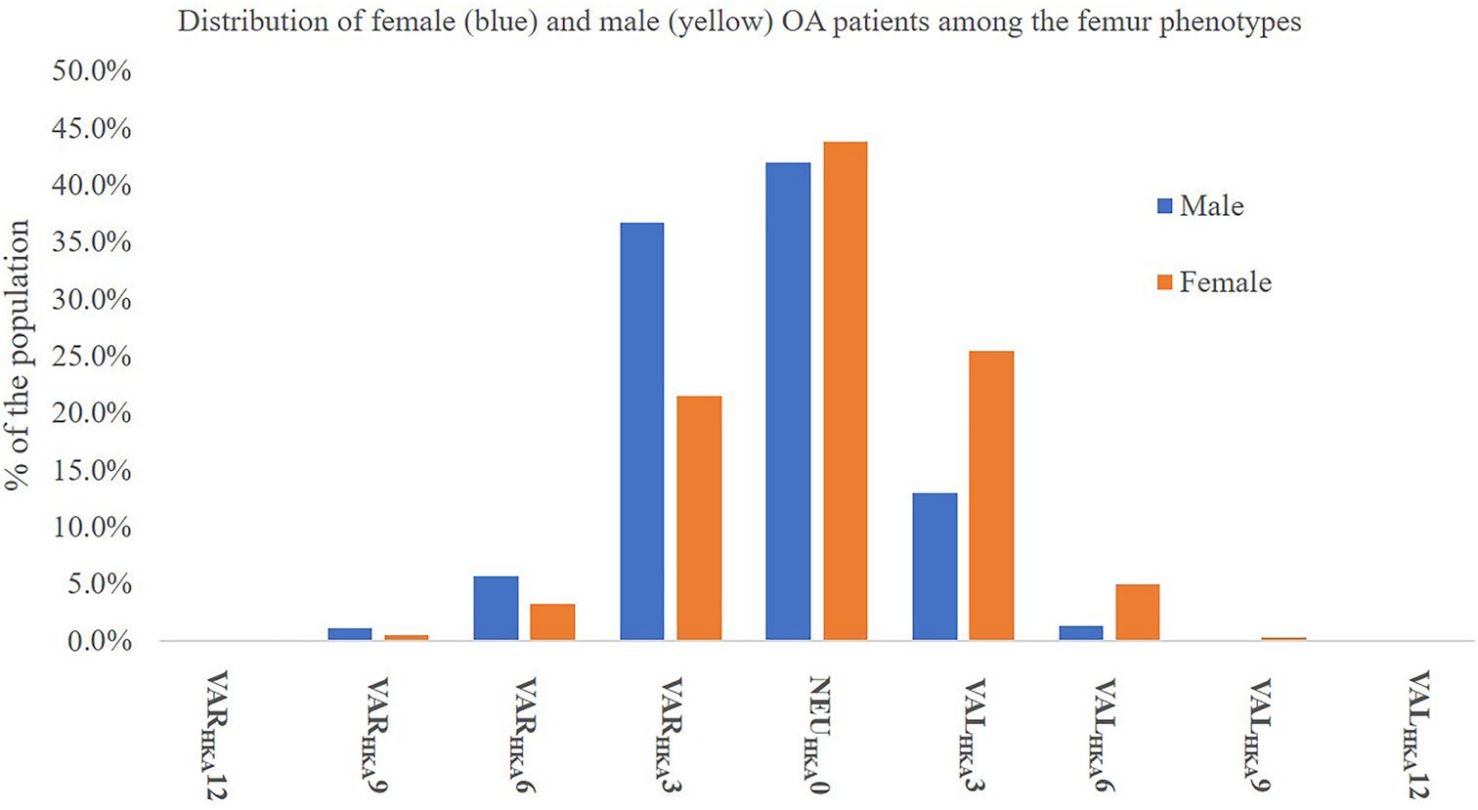
Distribution of the female (yellow) and male (blue) OA population among the femur phenotypes in %

**Fig. 4:**
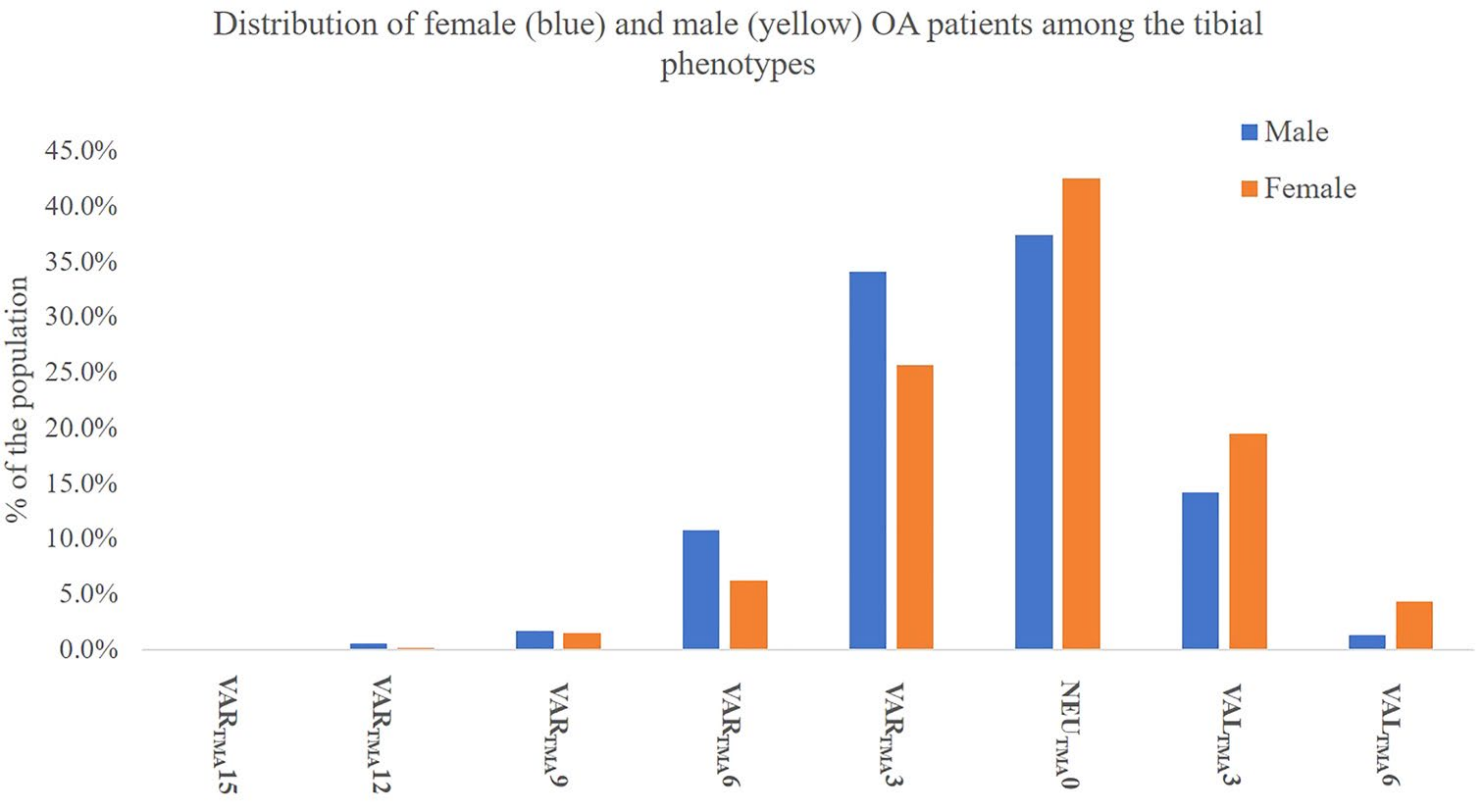
Distribution of the female (yellow) and male (blue) population among the tibial phenotypes in %

The number and distribution of limb, tibial and femoral phenotypes are shown in Figs. 2, 3 and 4.

### Knee phenotypes

There were 45 different knee phenotypes (combinations of femoral and tibial joint line orientation), 39 in the female and 37 in the male population. Of these 45 knee phenotypes, 27 each covered less than 1% of the population. Table 2 shows the distribution among the knee phenotypes.

**Table 2:**
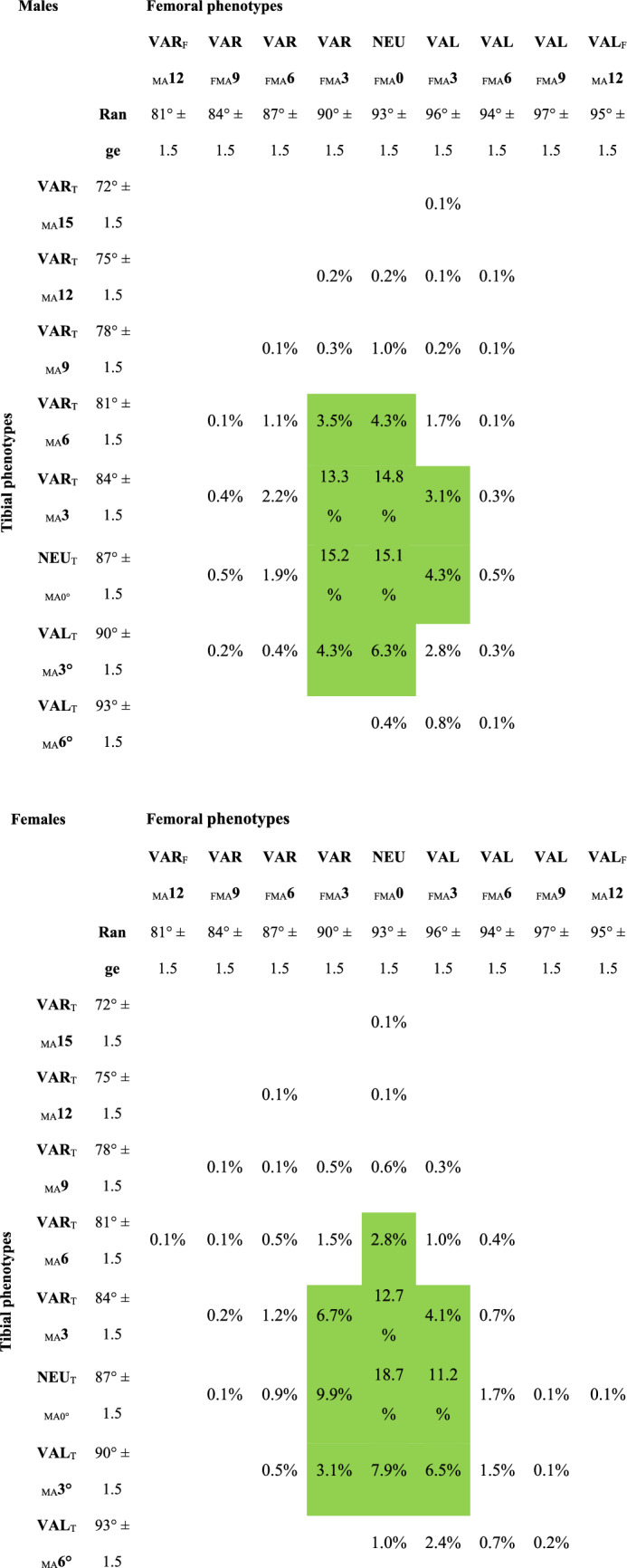
Distribution of male and female patients among the knee phenotypes

The green marked fields represent the most common knee phenotypes of each gender. They represent 84.1% and 83.5% of the male and female population respectively

### Functional knee phenotype of OA patients

There were 162 functional knee phenotypes and their distribution differed significantly between males and females. Separated by gender, there were 119 males, 136 females and 94 mutual phenotypes. The ten most common functional phenotypes in females and males are shown in Table 3. Out of the total 162 phenotypes, 46 only occurred once and 134 accounted each for less than 1% of the total population.

**Table 3:** The ten most common functional knee phenotypes of the male and female OA population

Males			
Rang	*N*	%	Functional knee phenotype
1	88	8.1	VAR_HKA_6° VAR_FMA_3° NEU_TMA_0°
2	80	7.4	VAR_HKA_6° NEU_FMA_0° VAR_TMA_3°
3	65	6	VAR_HKA_9° VAR_FMA_3° VAR_TMA_3°
4	60	5.6	VAR_HKA_3° NEU_FMA_0° NEU_TMA_0°
5	53	4.9	VAR_HKA_6° VAR_FMA_3° VAR_TMA_3°
6	47	4.4	VAR_HKA_6° NEU_FMA_0° NEU0°
7	44	4.1	VAR_HKA_3° NEU_FMA_0° VAR_TMA_3°
8	41	3.8	NEU_HKA_0°NEU_FMA_0° NEU_TMA_0°
9	38	3.5	VAR_HKA_9° VAR_FMA_3° NEU_TMA_0°
10	29	2.7	VAR_HKA_3° VAR_FMA_3° NEU_TMA_0°
Total	545	50.5	

Females			
Rang	*N*	%	Functional knee phenotype
1	147	13.6	VAR_HKA_3° NEU_FMA_0° NEU_TMA_0°
2	90	8.3	VAR_HKA_6° NEU_FMA_0° VAR_TMA_3°
3	80	7.4	VAR_HKA_6° VAR_FMA_3° NEU_TMA_0°
4	64	5.9	NEU_HKA_0°NEU_FMA_0°NEU_TMA_0°
5	62	5.7	VAL_HKA_3° VAL_FMA_3° NEU_TMA_0°
6	60	5.6	VAR_HKA_6° NEU_FMA_0° NEU_TMA_0°
7	51	4.7	VAR_HKA_3° NEU_FM_0° VAR_TMA_3°
8	49	4.5	VAR_HKA_9° VAR _FMA_3° VAR_TMA_3°
9	49	4.5	NEU_HKA_0° NEU_FMA_0° VAL_TMA_3°
10	48	4.4	NEU_HKA_0° VAL_FMA_3° NEU_TMA_0°
Total	700	64.80	

### Comparison of OA and non-OA population

Table 4 shows the comparison of alignment parameters between non-OA and OA patients separated by gender. To further assess these differences in mean values, additional subgroup analysis was performed for varus, neutral and valgus subgroups, separated by gender (Table 5). At this point, it seems important to note that the number of females for the varus and valgus subgroup analysis was very limited (only 12 varus non-OA female patients). The distribution of patients among the phenotypes (limb, femoral, tibial, knee and functional knee) differed significantly between OA and non-OA population (*p* < 0.005 for all comparisons).

**Table 4:**
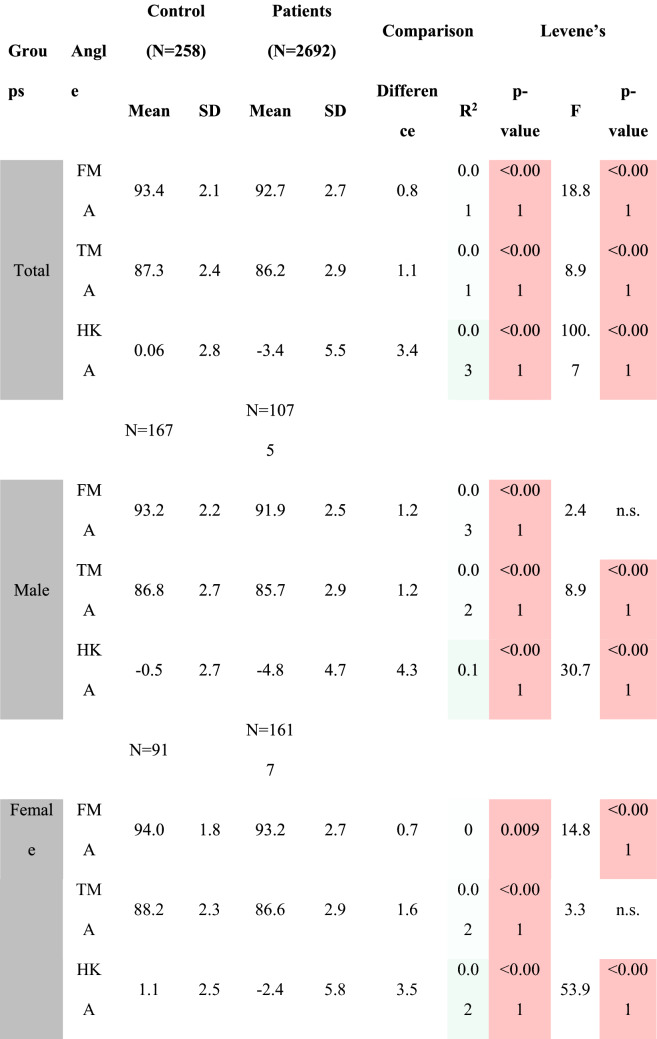
Comparison of alignment parameters between non-OA and OA patients separated by gender

Red marked fields show significant differences

**Table 5:**
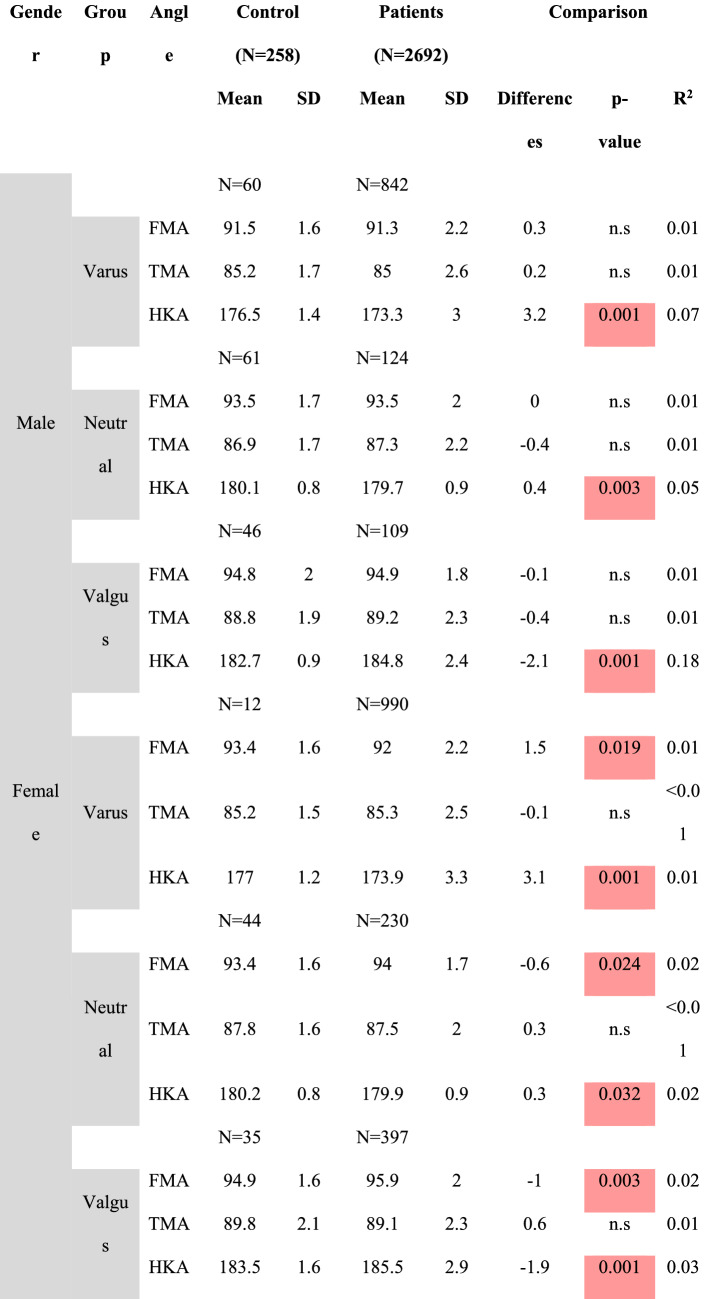
Comparison of alignment parameters between non-OA and OA patients separated by subgroups and gender

Red marked fields show significant differences

### Alignment concept

Total of 7.4% of all OA patients were excluded because their alignment would change from varus to valgus or vice versa. 36.3% of all OA patients had a functional knee phenotype within the boundaries. 30.1% and 45.7% would have needed an adaptation of their femoral and/or tibial joint line orientation respectively. 12.1% would need an adaptation of both, femoral and tibial joint line. Average change in HKA, FMA and TMA would have been 5.0° ± 3.1, 1.5° ± 1.4 and 2.0° ± 2.0. All patients would have had one of 6 postoperative phenotypes, which are shown in Table 6.

**Table 6:** Postoperative phenotypes of OA patients with the phenotype alignment concept

Postoperative phenotypes					
Rang	%	Limb (HKA)	Femur (FMA)	Tibia (TMA)	Ranges (HKA, FMA, TMA)
1	50.0	NEU_HKA_0°	NEU_FMA_0°	NEU_TMA_0°	180 ± 1.5, 93 ± 1.5, 87 ± 1.5
2	25.3	VAR_HKA_3°	VAR_FMA_3°	NEU_TMA_0°	177 + 1.5, 90 ± 1.5, 87 ± 1.5
3	12.0	VAL_HKA_3°	NEU_FMA_0°	VAL_TMA_3°	183 ± 1.5, 93 ± 1.5, 90 ± 1.5
4	6.4	NEU_HKA_0°	VAR_FMA_3°	NEU_TMA_0°	180 ± 1.5, 90 ± 1.5, 87 ± 1.5
5	4.5	NEU_HKA_0°	VAR_FMA_3°	VAL_TMA_3°	180 ± 1.5, 90 ± 1.5, 90 ± 1.5
6	1.8	NEU_HKA_0°	NEU_FMA_0°	VAL_TMA_3°	180 ± 1.5, 93 ± 1.5, 90 ± 1.5
Total	100.0				

## Discussion

The most important findings of the present study were the following:

First, the results of this study support our first hypothesis. Femoral and tibial joint line orientation varied within OA patients with the same overall lower limb alignment. Our results are supported by two recent studies. Sappey‑Marinier et al. described the alignment of 2859 OA patients based on weight-bearing long leg radiographic (LLR) measurements using the functional knee phenotype classification [17]. They excluded patients with severe bone loss (Ahlbäck classification > 3) and only reported femoral, tibial and knee phenotypes but not functional knee or limb phenotype. Their results are comparable to ours regarding femoral and tibial phenotypes but differ regarding the most common knee phenotypes, which might be attributed to a different gender distribution. In a very recent study MacDessi et al. used a different approach to define and assess joint line orientation but also reported variations in joint line orientation in patients with the same overall lower limb alignment [14].

Second, the results of this study are inconclusive regarding our second hypothesis. There were differences between non-OA and OA and patients, but they were small (< 2°) except for HKA. Furthermore, they did not reach significance when subgroups (e.g. only varus patients) were compared (except for HKA). Interpreting our results is difficult as there is only one comparable study based on 3D images and few studies based on LLR. Than et al. assessed the lower limb alignment of OA and non-OA patients using the EOS imaging system and found a significantly more varus-aligned tibial joint line in the OA population [18]. MacDessi et al. compared, among other parameters, the alignment of OA and no-OA patients based on LLR (500 non-OA and 500 OA patients) and found no difference in tibial and femoral joint line orientation but a significant difference in HKA [14]. Cooke et al. found a difference in the femoral joint line orientation between varus OA and non-OA patients and a difference in the tibial joint line orientation between valgus OA and non-OA patients in relatively small sample size [4]. Another interesting finding regarding the difference between OA and non-OA was reported by MacDessi et al. [15]. In a matched-pairs radiological study, they compared the calculated HKA (FMA + TMA) of an osteoarthritic knee with the measured HKA of the contralateral normal knee and found no difference. They concluded that the calculated HKA can be used to predict the constitutional alignment of a patient once arthritis has developed. Their results have some limitations since studies have found significant within-patient differences in alignment [2] and a change in the alignment of the non-affected knee [19]. Overall, current evidence indicates that there is only a small change in FMA and TMA with the development of OA (excluding cartilage loss) and that the pre-arthritic alignment might be estimated based on FMA and TMA.

This conclusion has several clinical consequences. First, our results support the ideas of some newer alignment concepts such as the pure and restricted kinematic alignment concept, which base their alignment goals on pre-or intraoperative measurements of FMA/TMA in the osteoarthritic situation [1, 11, 12]. Second, a comprehensive analysis of the alignment of patients scheduled for TKA seems essential because their alignment represents the foundation for an individualized alignment approach. More importantly, an adaptation of FMA and/or TMA in a majority of patients is necessary to avoid a severe varus or valgus TKA components alignment. Only 40% of our OA population (39.5% of all females, 40.8% of all males) would have had a postoperative FMA between 88.5° and 94.5° and a TMA between 86.5° and 91.5°, if FMA and TMA remained unchanged. The functional knee phenotype system thereby could be a useful tool since it provides a comprehensive and adaptable, yet simple way to assess the patient’s alignment and define alignment targets. These alignment targets would be specific for a group of patients with a similar alignment variation (e.g. phenotype) rather than for all patients. The example on how this could work, presented in this paper, is interesting for several reasons. First, the concept would result in three phenotypes in over 85% of all patients (NEU_HKA_0° NEU_FMA_0° NEU_TMA_0° = HKA 180° ± 1.5 FMA 93° ± 1.5 TMA 87° ± 1.5, VAR_HKA_3° VAR_FMA_3° NEU_TMA_0° = HKA 183° ± 1.5 FMA 90° ± 1.5 TMA 87° ± 1.5, VAL_HKA_3°NEU_FMA_0°VAL_TMA_3° = HKA 177° ± 1.5 FMA 93° ± 1.5 TMA 90° ± 1.5). Second, these three phenotypes all represent previously established alignment variations. The majority of patients (50%) would end up with an anatomical alignment, which has been found to produce good clinical results and long-term survivorship [21]. Another 25% would have an slight varus alignment (mechanically neutral femoral component and varus tibia), which might result in superior clinical outcomes in varus patients compared to the mechanical alignment concept [20]. Twelve per cent of all OA patients would have a slight valgus alignment (valgus femur and a mechanically neutral tibia). Based on our clinical experience, this postoperative alignment would be acceptable for most surgeons in a preoperative valgus patient.

Several limitations must be acknowledged for this study. First, it has a selection bias as not all OA patients are represented in the Knee-PLAN^®^ 3D database and not all patients receive a CT scan. The database was established with patients undergoing planning for TKA, so only progressed OA patients have been included in this study. However, this is one of the largest samples investigated and the ranges found in this study were comparable to previously reported ranges. Besides, the large sample size reduces this risk further. Secondly, OA grades were not available for assessment and it has been shown that the grade of OA influences overall alignment [19]. Thirdly, our knowledge regarding patient characteristics was limited to age, gender and information visible on CT images. Fourthly, our measurements are based on supine CT images and therefore the influence of weight-bearing/laxity could not be assessed. In fact, the influence of laxity only has relevance for HKA measurements as TMA and FMA are not influenced by weight bearing. Finally and most importantly, as this is a cross-sectional study the comparison between OA and non-OA patients needs to be interpreted with all due caution.

Despite this limitation, our work might help surgeons better understand the impact of new alignment concepts and the reasoning behind them. Last but not least, surgeons should be aware that FMA and TMA need to be adapted in somewhat to achieve a postoperative alignment within certain limits. Further research will be necessary to define how much change is needed.

## Conclusion

Differences between OA and non-OA knees are small regarding coronal femoral and tibial joint line orientation. Femoral and tibial joint line orientation of osteoarthritic patients can, therefore, be used to estimate their native coronal alignment and plan individualized alignment.

## Abbreviations

CT: Computed tomography
FMA: Femoral mechanical angle
HKA: Hip-knee-ankle angle
HDP: Hydroxymethane diphosphonate
NEU: Neutral
Non-OA: Non-osteoarthritis
OA: Osteoarthritis
SD: Standard deviation
SPECT: Single photon emission computed tomography
TMA: Tibial mechanical angle
TKA: Total knee arthroplasty
VAL: Valgus
VAR: Varus
